# Trait-anxiety among healthcare professionals in COVID-19 pandemic

**DOI:** 10.1192/j.eurpsy.2021.1794

**Published:** 2021-08-13

**Authors:** A. Bouaziz, N. Smaoui, M. Kraiem, R. Feki, S. Omri, M. Maalej Bouali, N. Charfi, L. Zouari, J. Ben Thabet, M. Maalej

**Affiliations:** 1 Psychiatry C Departement, Hospital University of HEDI CHAKER, sfax, Tunisia; 2 Psychiatry C Department, Hedi chaker University hospital, sfax, Tunisia; 3 Psychiatry C, Hedi Chaker University Hospital, sfax, Tunisia

**Keywords:** Healthcare professionals, Trait-Anxiety, Associated factors, Covid-19 pandemic

## Abstract

**Introduction:**

Factors causing anxiety among healthcare professionals (HCP) are increasing, and psychosocial causes are the most common. During the COVID-19 pandemic, HCP are exposed to additional stressful factors.

**Objectives:**

The aim of this study was to assess the prevalence of trait-anxiety and its associated factors towards the COVID-19 outbreak among Tunisian HCP.

**Methods:**

A cross-sectional descriptive and analytical study conducted among Tunisian HCP during November and December 2020. The data were collected by an online questionnaire including the sociodemographic information and the “General anxiety questionnaire of Spielberger” (STAI-Y-B) which was used to assess the trait-anxiety.

**Results:**

A total of 135 HCP participated in this study (47.4% female and 52.6% male). The average age was 31.98 years (SD=6.59). Of the participants, 3% were nurses, 8.1% were interns, 48.1% were residents, 34.8% were specialist doctors and 5.9% were generalist doctors. Concerning marital status, 61.5% were single, 36.3% were married and 2.2% were divorced. Of HCP involved in the study, 13.3% of the participants had a history of chronic somatic-disorder and 11.9 % of them had a history of a psychiatric disorder. The prevalence of trait-anxiety in HCP was 53.3%. The analysis showed that anxious HCP were more younger (p=0.002) and had history of a psychiatric disorder (p=0.017) compared to non-anxious HCP. However, no significant difference was found by gender, marital status and professional degree according to trait-anxiety.

**Conclusions:**

In our study, we found that more than the half of HCP were anxious and these one were more younger and had history of psychiatric disorders.

**Disclosure:**

No significant relationships.

